# Gα_16 _interacts with tetratricopeptide repeat 1 (TPR1) through its β3 region to activate Ras independently of phospholipase Cβ signaling

**DOI:** 10.1186/1472-6807-11-17

**Published:** 2011-04-13

**Authors:** Andrew MF Liu, Rico KH Lo, Emily X Guo, Maurice KC Ho, Richard D Ye, Yung H Wong

**Affiliations:** 1 Division of Life Science and the Biotechnology Research Institute, Hong Kong University of Science and Technology, Clear Water Bay, Kowloon, Hong Kong, China; 2 State Key Laboratory of Molecular Neuroscience, and the Molecular Neuroscience Center, Hong Kong University of Science and Technology, Clear Water Bay, Kowloon, Hong Kong, China; 3 Department of Pharmacology, College of Medicine, University of Illinois, Chicago, Illinois 60612, USA; 4 Cell and Molecular Biology Programme, School of Life Sciences, The Chinese University of Hong Kong, Hong Kong, China; 5 Department of Medicine, The University of Hong Kong, Hong Kong, China

## Abstract

**Background:**

G protein-coupled receptors constitute the largest family of cell surface receptors in the mammalian genome. As the core of the G protein signal transduction machinery, the Gα subunits are required to interact with multiple partners. The GTP-bound active state of many Gα subunits can bind a multitude of effectors and regulatory proteins. Yet it remains unclear if the different proteins utilize distinct or common structural motifs on the Gα subunit for binding. Using Gα_16 _as a model, we asked if its recently discovered adaptor protein tetratricopeptide repeat 1 (TPR1) binds to the same region as its canonical effector, phospholipase Cβ (PLCβ).

**Results:**

We have examined the specificity of Gα_16_/TPR1 association by testing a series of chimeras between Gα_16 _and Gα_z_. TPR1 co-immunoprecipitated with Gα_16 _and more tightly with its constitutively active Gα_16_QL, but not Gα_z_. Progressive replacement of Gα_16 _sequence with the corresponding residues of Gα_z _eventually identified a stretch of six amino acids in the β3 region of Gα_16 _which are responsible for TPR1 interaction and the subsequent Ras activation. Insertion of these six residues into Gα_z _allowed productive TPR1-interaction. Since the β3 region only minimally contributes to interact with PLCβ, several chimeras exhibited differential abilities to stimulate PLCβ and Ras. The ability of the chimeras to activate downstream transcription factors such as signal transducer and activator of transcription 3 and nuclear factor κB appeared to be associated with PLCβ signaling.

**Conclusions:**

Our results suggest that Gα_16 _can signal through TPR1/Ras and PLCβ simultaneously and independently. The β3 region of Gα_16 _is essential for interaction with TPR1 and the subsequent activation of Ras, but has relatively minor influence on the PLCβ interaction. Gα_16 _may utilize different structural domains to bind TPR1 and PLCβ.

## Background

Heterotrimeric guanine nucleotide-binding proteins (G proteins) are multifaceted signaling modules that relay extracellular signals detected by G protein-coupled receptors (GPCRs) to intracellular effector [1-3]. At the core of the G protein signal transduction machinery is the Gα subunit, a GTPase which acts as a timer to limit the activation signal. In the classical G protein activation cycle, the Gα subunit needs to associate with the Gβγ dimer, the GPCR, and effectors separately or simultaneously at different stages of the cycle. A variety of accessory proteins are now known to modulate the fidelity of the G protein signal. They include regulators of G protein signaling (RGS) [4], activators of G protein signaling (AGS) [5], and adaptor proteins such as tetratricopeptide repeat 1 (TPR1) [6]. These additional components allow for rapid inactivation or receptor-independent activation of the Gα subunit, as well as signal diversification. The large number of different types of binding partners for the Gα subunit requires optimal utilization of structural domains that are available for protein-protein interactions. Given that Gα subunits are typically less than 50 kDa in size and are attached to the inner leaflet of the plasma membrane, the binding surfaces available for interaction are limited. Nature has partially resolved this constraint by generating different conformations of the Gα subunit through the binding and hydrolysis of GTP.

**Figure 1:**
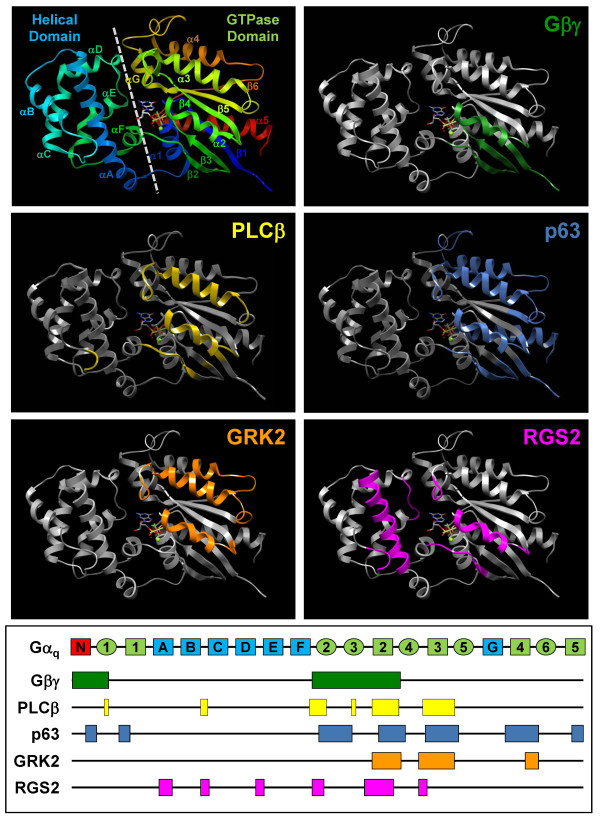
**Structural representations of the functional domains of Gα_q_**. The crystal structure of Gα_q _(based on the complex with p63RhoGEF and RhoA, PDB ID: 2rgnA) is depicted with the different functional domains highlighted. The top left panel is the Gα_q _structure in rainbow colors (blue to red as from N- to C-terminus). Nomenclature of α helices and β strands is according to the first resolved Gα crystal structure [19]. The dotted line divides the structure into two parts, known as the helical and GTPase domains. The other five structures are shown in the same orientation as the top left panel, with the putative interacting domains for Gβγ (green), PLCβ (yellow), p63RhoGEF (p63, light blue), GRK2 (orange) and RGS2 (magenta) highlighted. Mapping of the various interacting domains are based on resolved crystal structures (for Gβγ, PLCβ, p63RhoGEF and GRK2) or structural alignment with other Gα subunit complex structures (for RGS2 in complex with Gα_i3_). The bottom panel is a simplified linear representation of Gα_q _with the secondary structures belonging to helical (light blue) and GTPase domains (light green) highlighted; α-helices and β-strands are depicted as rectangles and ovals, respectively. The N-terminal helix colored in red remains unresolved in the crystal structure of Gα_q_. The corresponding functional domains of the five interacting partners as shown in the molecular models above the schematic are indicated with the same color scheme.

The resolution of the crystal structures of several Gα subunits in their GDP- or GTP-bound states [7,8] and as complexes with the Gβγ dimer [9-11] or other interacting proteins [12-17] have provided valuable insight into the molecular mechanisms of G protein signal transduction. Structurally, the Gα subunit can be broadly divided into the GTP hydrolase (GTPase) and helical domains (Figure 1) with the former harboring the GTP-binding pocket [18,19]. Several regions (Switch I-IV) spreading across the GTPase and helical domains exhibit profound conformational changes when the Gα subunit shifts between the GDP- and GTP-bound states [20,21]. Changes in the switch regions provide the molecular basis of G protein activation and effector regulation. In the GTP-bound active state, the Gα subunit releases the Gβγ dimer and thus allows effectors to bind to the newly exposed surfaces such as the Switch II region [20,21]. This simplistic view, however, cannot accommodate the increasing numbers of Gα-interacting proteins. The activated Gα subunit is a preferred partner for multiple effectors, adaptors, and RGS proteins. A central question is whether an activated Gα subunit can concurrently regulate multiple signaling pathways by simultaneously binding to different partners in much the same way as an inactive Gα subunit forms a complex with the Gβγ dimer and the receptor.

Among the different subfamilies of Gα subunits, members of the Gα_q _subfamily have the capacity to activate phospholipase Cβ (PLCβ) [22,23] as well as interact with the guanine nucleotide exchange factor p63RhoGEF [24,25], G protein-coupled receptor kinase GRK2 [26-28], adaptor proteins such as TPR1 [6], and several RGS proteins [29-32]. These molecules bind to overlapping as well as distinct regions on Gα_q _(Figure 1). It is often assumed that the primary signal generated by G_q_-coupled receptors is the formation of inositol trisphosphates (IP_3_) by PLCβ, and that the regulation of downstream kinases and transcription factors are consequential to the production of IP_3 _and the subsequent Ca^2+ ^mobilization. However, recent studies suggest that Gα subunits can concurrently regulate multiple signaling pathways. The ability of Gα_16_, a member of the Gα_q _subfamily, to interact with the adaptor protein TPR1 [6] has raised some interesting scenarios. Gα_16 _is primarily expressed in hematopoietic cells and it can regulate multiple signaling pathways [25,33]. Interestingly, TPR1 can directly interact with Ras especially when the latter is activated [6] and it appears to link Gα_16 _to Ras activation [34]. Ras is a small GTPase which acts as a molecular switch for linking various cell surface receptors to intracellular signaling pathways, resulting in cell proliferation and differentiation [35]. We have recently demonstrated that constitutively active Gα_16 _(Gα_16_QL) induces the phosphorylations of transcription factors, such as signal transducer and activator of transcription 3 (STAT3) and nuclear factor κB (NFκB), through PLCβ and Ras/Raf-1/MEK/ERK signaling cascades in human embryonic kidney 293 (HEK 293) cells [36,37]. However, it is not known if the binding of TPR1 to Gα_16 _affects PLCβ signaling and whether TPR1 and PLCβ utilize the same docking site on Gα_16_. Although Gα_16 _has been shown to interact with the C-terminus of TPR1 [6], the structural requirement for Gα_16 _to interact with TPR1 has yet to be defined. In the present study, we examined the structural domain of Gα_16 _for interacting with TPR1 and assessed whether the same domain is responsible for regulating PLCβ.

## Methods

### Materials

The human cDNAs of Gα_16_, Gα_16_QL, Gα_z _and Gα_z_QL were obtained from Missouri S&T cDNA Resource Center. C25 and C44 cDNAs were previously constructed and characterized [38]. Cell culture reagents, including Lipofectamine™ and Plus™ reagents, and AccuPrime™ *Pfx *SuperMix were purchased from Invitrogen (Carlsbad, CA). Anti-Gα_16 _(N-terminus) and anti-Gα_z _(C-terminus) were obtained from Gramsch Laboratories (Schwabhausen, Germany). Anti-Gα_16 _(C-terminus) was purchased from Torrey Pines Biolabs (East Orange, NJ). Anti-Gα_z _(N-terminus) and PLCβ antibodies were from Santa Cruz Biotechnology (Santa Cruz, CA). Unbound and affinity gel-conjugated anti-FLAG antibody were from Sigma-Aldrich (St. Louis, MO). Other antibodies were purchased from Cell Signaling Technology (Danvers, MA). Protein G-agarose and protein cross-linking agent dithiobis[succinimidylpropionate] were from Pierce Biotechnology (Rockford, IL). ECL kit was from GE Healthcare Bio-Sciences (Piscataway, NJ). Ras activation kit was a product of Millipore (Billerica, MA).

### Construction of chimeras

Gα chimeras were constructed from the cDNAs encoding the human Gα_16 _and Gα_z _by using PCR techniques. The N-terminal 102, 155, 188, 200, 210, 246, 266 and 295 residues of Gα_16 _were substituted by the corresponding regions of Gα_z _to generate N102, N155, N188, N200, N210, N246, N266 and N295 chimeras, respectively. Primer pairs were designed to cover the overlapping regions in forward and reversed directions. For each construct, the 5' fragment was generated with the reversed and T7 primers, whereas the 3' fragment was made with the forward and SP6 primer. The two half-products were then annealed together to generate a full-length fragment by another round of PCR using T7 and SP6 primers. Mirror-images of these constructs were generated analogously and were named C164, C174, C186, C219 and C272 chimeras. Primers for chimera construction are listed in Table 1. PCR was carried out using AccuPrime™ *Pfx *SuperMix (30 cycles each with 94°C for 60 s, 58°C for 60 s and 72°C for 90 s). The N200-C164 and N188-C164 chimeras were constructed using C164 as the initial template for the 3' half-products and those primers designed for N200 and N188, respectively. Likewise, zβ3 and zβ2β3 were constructed using the C174 and C164 primers with N210 as the initial templates. All chimeras were checked by restriction mapping and then subcloned into pcDNA3 at *Hind *III and *Xho *I sites. The constructs were fully sequenced by dideoxynucleotide sequencing to confirm the identities.

**Table 1:** Primer sequences for constructing chimeras between Gα_16 _and Gα_z_.

*Construct*	*Primers:*	*Antisense Primer Sense Primer*	*Size (aa)*
N102	5'-GGGCCTGCTGAATGG ***GATCCTGAGGGCGGC ***-3'		365
	3'-CCCGGACGACTTACC***CTAGGACACCCGCCG***-5'		
N155	5'-GGCTGAATCGAGCAG***GTGGTACTCGCTGGA***-3'		367
	3'-CCGACTTAGCTCGTC***CACCATGAGCGACCT***-5'		
N188	5'-GTTGATGCCAGTGGT***CATGTCCCGGGAGCG***-3'		367
	3'-CAACTACGGTCACCA***GTACAGGGCCCTCGC***-5'		
N200	5'-CCGCAGGTTGGTTTT***CTTGAAGGTGAACTT***-3'		367
	3'-GGCGTCCAACCAAAA***GAACTTCCACTTGAA***-5'		
N210	5'-CTCTGACTTCTGGCC***CCCCACGTCCACCAT***-3'		367
	3'-GAGACTGAAGACCGG***GGGGTGCAGGTGGTA***-5'		
N210QL	5'-CTCTGACTTCAGGCC***CCCCACGTCCACCAT***-3'		367
	3'-GAGACTGAAGTCCGG***GGGGTGCAGGTGGTA***-5'		
N246	5'-CATGCGGTTCTCCTG***GTTATCCTCGTAGAG***-3'		367
	3'-GTACGCCAAGAGGAC***CAATAGGAGCATCTC***-5'		
N266	5'-GGATGTGCTTTTGAA***CCAGTTGTTGTTGCA***-3'		367
	3'-CCTACACGAAAACTT***GGTCAACAACAACGT***-5'		
N295	5'-GCCCTGGAAACTGGG***AAAGCAGATGGTGAG***-3'		367
	3'-CGGGACCTTTGACCC***TTTCGTCTACCACTC***-5'		
C164	5'-***CTCTGACCTCTGCCC***CCCGACGTCCACGAT-3'		362
	3'-***GAGCAYGGAGACGGG***GGGCTGCAGGTGCTA-5'		
C164QL	5'-***CTCTGACCTCAGCCC***CCCGACGTCCACGAT-3'		362
	3'-***GAGCATGGAGTCGGG***GGGCTGCAGGTGCTA-5'		
C174	5'-***CTTGAAGGTGAGCTC***CTGCACGGAGAAGCA-3'		362
	3'-***GAACTTCCACTCGAG***GACGTGCCTCTTCGT-5'		
C186	5'-***CACAATGCCCGTGGT***GGGCATGCGGCTGCG-3'		362
	3'-***GTGTTACGGGCACCA***CCCGTACGCCGACGC-5'		
C219	5'-***CGCGTTGTCCTCCAG***GTGGAATTCCCGCCG-3'		362
	3'-***GCGCAACAGGTCCAC***CACCTTAAGGGCGGC-5'		
C272	5'-***CTTGATTTCGCGGCG***GTGGTCCTGCTTCTG-3'		362
	3'-***GAACTAAAGCGCCGC***CACCAGGACGAAGAC-5'		

Bold and italic nucleotides denote the Gα_16_-derived sequences.

### Cell culture and co-immunoprecipitation experiments

HEK 293 cells were obtained from the American Type Culture Collection (CRL-1573, Rockville, MD). They were maintained in Eagle's minimum essential medium at 5% CO_2_, 37°C with 10% fetal bovine serum (FBS), 50 units/ml penicillin and 50 μg/ml streptomycin. HEK 293 cells were grown to 80% confluency in 100-mm tissue culture plates and then co-transfected with 800 ng Gα and 800 ng FLAG-TPR1 cDNAs using 15 μl Plus™ and Lipofectamine™ reagents in Opti-MEM™. FBS was replenished 3 h after transfection. Cross-linking was performed one day after transfection. Transfected cells were washed with PBS twice and then treated with 0.5 mM dithiobis[succinimidylpropionate] in PBS for 15 min at room temperature. Cells were then washed again with PBS and maintained in quenching solution (50 mM glycine in PBS, pH 7.4) for 5 min. Subsequently, cells were lysed in ice-cold RIPA buffer (25 mM HEPES at pH 7.4, 0.1% SDS, 1% Igepal CA-630, 0.5% sodium deoxycholate, 1 mM dithiothreitol, 200 μM Na_3_VO_4_, 4 μg/ml aprotinin, 100 μM phenylmethylsulfonyl fluoride, and 2 μg/ml leupeptin). Cell lysates were gently rocked with an anti-Gα_16_, anti-Gα_z _or anti-PLCβ antiserum at 4°C overnight, and then incubated in 30 μl protein G-agarose (50% slurry) at 4°C for 2 h. Alternatively, the cell lysates were incubated in 30 μl anti-FLAG affinity agarose gel (50% slurry) at 4°C overnight. Immunoprecipitates were washed with ice-cold RIPA buffer (400 μl) for four times, resuspended in 50 μl RIPA buffer and 10 μl 6 × sample buffer and then boiled for 5 min. Gα_16_, Gα_z_, PLCβ and FLAG-TPR1 proteins in the immunoprecipitates were analyzed by Western blots.

### Ras activation assay

HEK 293 cells were co-transfected with 800 ng Gα, 800 ng FLAG-TPR1 and 400 ng Ras cDNAs. After 1 day, transfectants were serum-deprived for 4 h. Cells were then washed twice with ice-cold PBS and lysed with the 1 × Mg^2+ ^lysis buffer (MLB: 25 mM HEPES at pH 7.5, 150 mM NaCl, 1% Igepal CA-630, 10 mM MgCl_2_, 1 mM EDTA, 2% glycerol, 200 μM Na_3_VO_4_, 4 μg/ml aprotinin and 2 μg/ml leupeptin). Cell lysates were pre-cleaned with Protein G-agarose and activated Ras was immunoprecipitated with 20 μl Raf-1 Ras-binding domain-conjugated agarose (Millipore) for 45 min and followed by three washes of 400 μl ice-cold MLB. Immunoprecipitates was finally reconstituted in 50 μl MLB and 10 μl 6 × sample buffer and resolved in SDS-PAGE for detecting Ras using specific antibody.

### Assays for phosphorylated ERK, IKK and STAT3

HEK 293 cells were seeded on a 6-well plate at 4.5 × 10^5 ^cells/well 1 day prior to transfection. Transfection was performed with 200 ng Gα and 200 ng FLAG-TPR1 cDNAs using 4 μl Plus™ and Lipofectamine™ reagents in Opti-MEM™. The transfectants were serum-deprived overnight one day after transfection. Cells were treated with pertussis toxin (PTX; 100 ng/ml for 16 h) and *N*^6^-cyclohexyladenosine (CHA; 10 μM for 15 min) where appropriate, lysed, and then assayed for phosphorylation statuses of ERK, STAT3, and IKK as described previously [36,37].

### Inositol trisphosphate accumulation assay

HEK 293 cells were seeded on a 12-well plate at 2 × 10^5 ^cells/well one day prior to transfection. Cells were transfected with 300 ng Gα with or without 200 ng type 1 adenosine receptor (A_1_R) cDNAs using 2 μl Lipofectamine™ 2000 reagent in Opti-MEM™ containing 5% FBS. Transfectants were labeled with 2.5 μCi/ml *myo*-[^3^H]inositol and subsequently assayed for CHA-induced [^3^H]IP_3 _formation as described previously [39].

### NFκB-driven and STAT3-driven luciferase assays

For STAT3-driven luciferase assay [40], HEK 293 cells were seeded on a 96-well microplate at 10,000 cells/well one day before transfection. Cells were transfected with 10 ng Gα and 100 ng pSTAT3-luc luciferase reporter using 0.2 μL Plus™ and Lipofectamine™ reagents in Opti-MEM™ and FBS was replenished after 3 h. When A_1_R-induced signals were analyzed, 10 ng receptor cDNA was included in the transfection. For NFκB-driven luciferase assay, HEK 293 cells stably transfected with pNFκB-luc luciferase reporter were seeded on a 96-well microplate at 15,000 cells/well. The setup and transfection were as described previously [36]. One day after transfection, transfectants were serum-deprived for 4 h and PTX (100 ng/ml) was added where necessary. Cells were challenged with or without 10 μM CHA for 16 h before measuring the luciferase activity as reported previously [36].

### Western blotting analysis

Protein samples were resolved on 12% SDS-PAGE and transferred to nitrocellulose membrane (GE Osmonics). Resolved proteins were detected by their specific primary antibodies and horseradish peroxidase-conjugated secondary antisera. The immunoblots were visualized by chemiluminescence with the ECL kit from GE Healthcare Bio-Sciences, and the images detected in X-ray films were quantified by densitometric scanning using the Eagle Eye II gel documentation system (Stratagene, La Jolla, CA, USA).

### Molecular modeling of Gα subunits

Gα_q _in the complex with p63RhoGEF and RhoA (PDB ID: 2rgnA) [13] was employed for the illustration of functional domains of Gα_q_, and for creating a molecular model of Gα_16 _by homologous modeling using SWISS-MODEL [41], which allowed manual adjustments to the alignment of the sequences of Gα_16 _with Gα_q _in order to accommodate the extraordinarily long α4/β6 loop of Gα_16_. Molecular models of Gα_z _with or without the mutations at β2/β3 loop (^194^ELTFKM → KTNLRI) were generated using 3D-JIGSAW [42] based on the crystal structure of Gα_i1 _bound to AlF_4_^- ^and GDP (PDB ID: 2hlbA) [43] as selected by the default automatic mode. 3D-JIGSAW-generated models showed greater dynamics in the loop structures and allowed for the exploration of potential conformational variations caused by the mutations. Visualization of various structures was accomplished using UCSF Chimera [44].

## Results

### Extreme termini of Gα_16 _are not involved in TPR1 interaction

**Figure 2:**
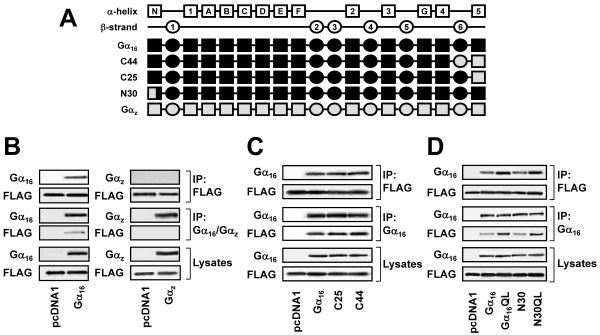
**Extreme termini of Gα_16 _are not required for TPR1 interaction**. (A) Schematic representation of the C25, C44, and N30 chimeras. Predicted secondary structures are illustrated as boxes (α helices) or ovals (β strands) above the chimeras. Closed areas represent human Gα_16 _sequence while those in open shapes signify the corresponding sequence of human Gα_z_. (B-D) HEK 293 cells were transiently co-transfected with FLAG-TPR1 and the wild-type or constitutively active mutants of Gα_16_, Gα_z_, C25, C44, N30, or pcDNA1. Cell lysates from the transfectants were immunoprecipitated (IP) by anti-FLAG affinity agarose gel (upper panels), anti-Gα_16 _or anti-Gα_z _antiserum (middle panels). The immunoprecipitates were immunoblotted with anti-Gα_16_, anti-Gα_z_, or anti-FLAG antiserum. Aliquots of cell lysates were used to detect the expression levels of Gα_16_, Gα_z _and FLAG-TPR1 by western blot analysis (lower panels). Data shown represent one of three sets of immunoblots; two other sets yielded similar results.

Unlike the receptor-interacting domain which is composed of five distinct structures [45-47], no discrete localization for effector interaction is generally applicable to the Gα subunits, probably because the different signaling pathways entail a diverse spectrum of effector molecules. The Gα_16 _regions responsible for effector interaction have not been mapped, but there is evidence to suggest that the Switch III and the α3 helix may participate in the binding of p63RhoGEF [48] and that the PLCβ-interacting domain on Gα_q _mainly encompasses the α2-β4-α3-β5 regions [49]. The involvement of β2 strand, α2 and α3 helices in the Gα_q_/PLCβ complex formation has been revealed clearly in the very recently resolved crystal structure [50] which also confirmed that PLCβ can serve as a GAP for Gα_q _[51]. In order to identify the structural domains on Gα_16 _that interact with TPR1, Gα_16 _sequences were progressively replaced by those of Gα_z _because the latter does not recognize TPR1 (Figure 2B). Chimeras composed of Gα_16 _and Gα_z _residues were preferred because they are structurally viable [38]. A series of chimeras was made by swapping discrete regions between Gα_16 _and Gα_z_. Construction of the chimeras was guided by the predicted tertiary structure of the Gα subunits as well as by our previous experience in determining the receptor and effector interacting domains of Gα_16 _and Gα_z _[38,52-55]. For chimeras with substitutions at either the N- or C-terminus, they were named with a single letter (N or C) followed by the numbers of amino acids of Gα_z _that substituted the corresponding regions of Gα_16_. The two chimeras containing either β2 or β2-β3 strands of Gα_z _were named with the a letter "z" followed by "β2" or "β2β3", respectively, in order to distinguish them from the descriptions of specific β strand structures.

We first examined two chimeras of Gα_16 _containing either 25 or 44 amino acids of Gα_z _at the C-terminus; representing changes in the α5 helix alone and α4/β6 loop plus the α5 helix, respectively. These two chimeras, named C25 and C44 respectively (Figure 2A), have been previously constructed and characterized (equivalent to the 16z25 and 16z44 of [38], respectively). Both chimeras exhibit enhanced coupling to G_i_-coupled receptors and possess the ability to stimulate PLCβ. HEK 293 cells were co-transfected with FLAG-TPR1 in combination with pcDNA1, Gα_16_, C25 or C44. As illustrated in Figure 2C (upper panels), Gα_16_, C25 and C44 were co-immunoprecipitated with the anti-FLAG affinity gel with similar levels of FLAG-TPR1. Moreover, FLAG-TPR1 was coimmunoprecipitated along with Gα_16_, C25 or C44 (Figure 2C, middle panels). Gα_16_, C25, C44 and FLAG-TPR1 were expressed at detectable and comparable levels in the transfectants (Figure 2C, lower panels). Since both chimeras retained the ability to interact with FLAG-TPR1, it implies that the C-terminal β6 and α5 regions of Gα_16 _are not required for the interaction with TPR1.

The N-terminus of Gα subunits participates in membrane attachment, Gβγ binding as well as receptor recognition. Another previously constructed chimera, N30 [38], was employed to test the possible involvement of the N-terminus of Gα_16 _in TPR1-binding. The αN helix (first 30 amino acids) of N30 is composed of Gα_z _sequence (Figure 2A). As shown in Figure 2D (upper panels), both Gα_16 _and N30 co-immunoprecipitated with FLAG-TPR1 and similar levels of FLAG-immunoreactivity were observed. Similarly, FLAG-TPR1 co-immunoprecipitated with Gα_16 _and N30 and the levels of these Gα subunits in the immunoprecipitates were essentially the same (Figure 2D, middle panels). Like Gα_16_QL, constitutively active N30QL was more readily associated with TPR1 (Figure 2D, upper and middle panels) and this effect was not due to variations in expression levels (Figure 2D, lower panels). Given that N30 still possessed the ability to interact with FLAG-TPR1, our results suggest that the region which is critical for direct or indirect binding of TPR1 may lie between the β1 strand and α4 helix of Gα_16_.

### The β3 region of Gα_16 _interacts with TPR1

**Figure 3:**
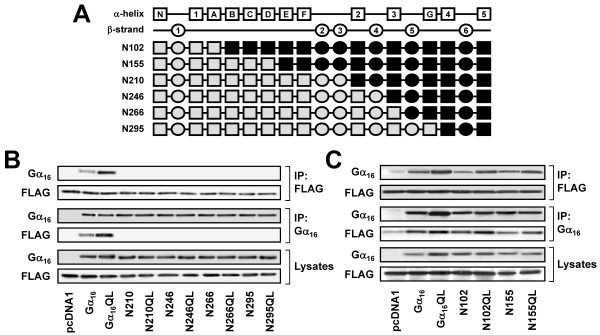
**Localization of the TPR1-interacting domain to the αE-αF-β2-β3 regions of Gα_16_**. (A) Schematic representation of the N102, N155, N210, N246, N266, and N295 chimeras. (B-C) HEK 293 cells were transiently co-transfected with FLAG-TPR1 and the wild-type or constitutively active mutants of Gα_16_, N102, N155, N210, N246, N266, N295, or pcDNA1. Co-immunoprecipitation assays were performed and analyzed as in Figure 2. Data shown represent one of three sets of immunoblots; two other sets yielded similar results.

To further examine the structural domain of Gα_16 _for TPR1 interaction, additional chimeras were constructed by replacing different regions of Gα_16 _with those of Gα_z_. Four different chimeras named N210, N246, N266 and N295 were thus constructed (Figure 3A) by replacing a Gα_z _backbone with Gα_16 _sequences starting from the α2, α3, β5, and α4 regions, respectively. However, none of the four Gα_16 _chimeras could be pulled down by anti-FLAG affinity gel (Figure 3B, upper panels). Similarly, FLAG-TPR1 could not be co-immunoprecipitated by anti-Gα_16 _antiserum in chimera-expressing cells (Figure 3B, middle panels) despite detectable expression levels of FLAG-TPR1 and the chimeras in the cell lysates (Figure 3B, lower panels). In control experiments, both Gα_16 _and Gα_16_QL were co-immunoprecipitated with FLAG-TPR1 (Figure 3B). These findings demonstrate that the TPR1-interacting domain must reside between residues 30 and 210 that represent the regions from β1 to β3 of Gα_16_.

Two more chimeras were constructed to map the TPR1-interacting domain within the first 30-210 residues. Chimeras N102 and N155 were made by replacing the N-terminus of Gα_16 _with Gα_z _sequences up to and including the αA and αD regions, respectively (Figure 3A). Both N102 and N155 were found to co-immunoprecipitate with FLAG-TPR1 (Figure 3C, upper panels). Association of these two chimeras with FLAG-TPR1 was confirmed by reverse co-immunoprecipitation using the anti-Gα_16 _antiserum (Figure 3C, middle panels). These results narrowed down the TPR1-interacting domain of Gα_16 _to be among the αE to β3 regions.

**Figure 4:**
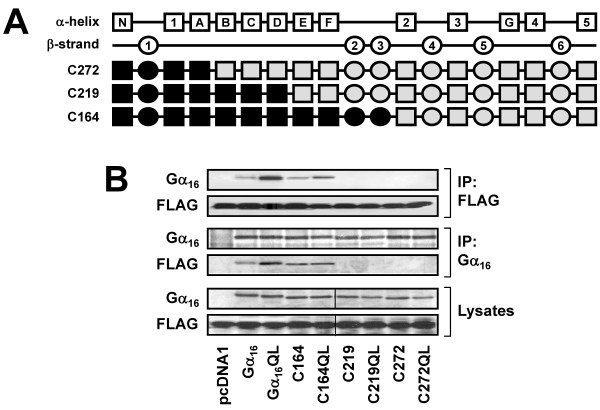
**Localization of the TPR1-interacting domain to the αE-αF-β2-β3 regions of Gα_16_**. (A) Schematic representation of the C272, C219, and C164 chimeras. (B) HEK 293 cells were transiently co-transfected with FLAG-TPR1 and the wild-type or constitutively active mutants of Gα_16_, C272, C219, C164, or pcDNA1. Co-immunoprecipitation assays were performed and analyzed as in Figure 2. Data shown represent one of three sets of immunoblots; two other sets yielded similar results.

**Figure 5:**
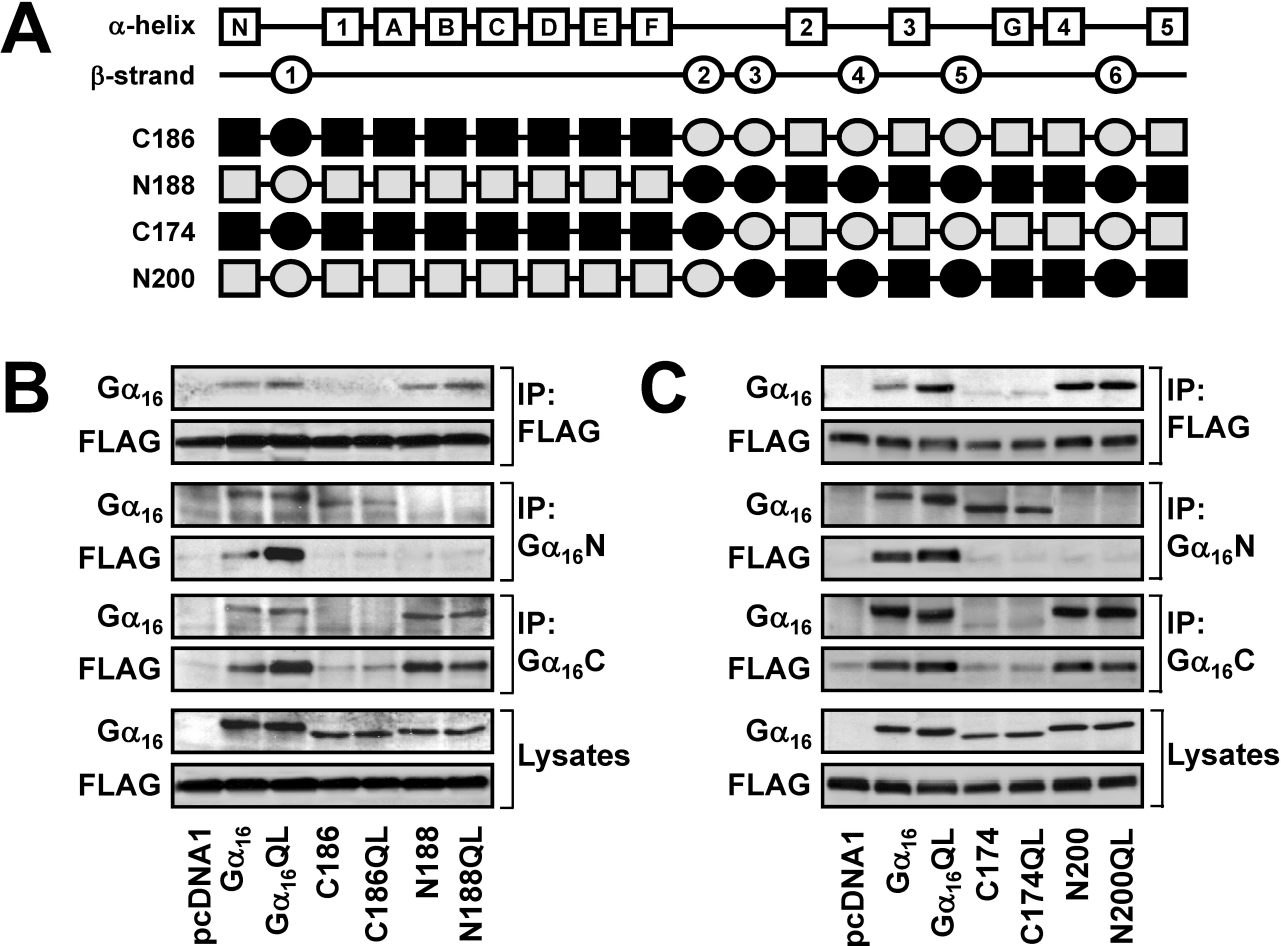
**Identification of the β3 region as the TPR1-interacting site of Gα_16_**. (A) Schematic representation of the C186, N188, C174, and N200 chimeras. (B-C) HEK 293 cells were transiently co-transfected with FLAG-TPR1 and the wild-type or constitutively active mutants of Gα_16_, C186, N188, C174, N200, or pcDNA1. Co-immunoprecipitation assays were performed and analyzed as in Figure 2. Data shown represent one of three sets of immunoblots; two other sets yielded similar results.

**Figure 6:**
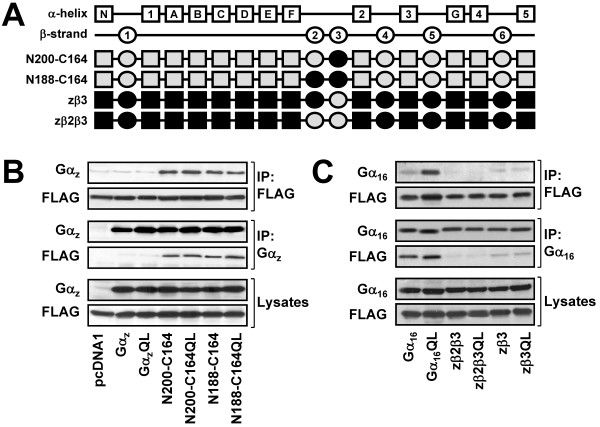
**Confirmation of the β3 region as a TPR1-interacting site of Gα_16_**. (A) Schematic representation of the N200-C164, N188-C164, zβ2β3, and zβ3 chimeras. (B-C) HEK 293 cells were transiently co-transfected with FLAG-TPR1 and the wild-type or constitutively active mutants of Gα_16_, N200-C164, N188-C164, zβ2β3, zβ3 or pcDNA1. Co-immunoprecipitation assays were performed and analyzed as in Figure 2. Data shown represent one of three sets of immunoblots; two other sets yielded similar results.

Before pinpointing the precise location of the TPR1-interacting site on Gα_16_, we first confirmed the preceding observations with additional chimeras that represent the mirror images of several tested chimeras. Chimeras C272, C219, and C164 were essentially the mirror images of N102, N155, and N210, respectively (Figure 4A), and they should exhibit phenotypes opposite to those of their counterparts. Indeed, C272 and C219 could not associate with FLAG-TPR1 even though they were efficiently expressed (Figure 4B). Conversely, chimera C164 should be able to interact with FLAG-TPR1 because its mirror image (N210) failed to associate with FLAG-TPR1; co-immunoprecipitation of C164 and FLAG-TPR1 confirmed their association (Figure 4B). These results again indicate that the TPR1-interacting domain of Gα_16 _lies within the αE-αF-β2-β3 regions which are common in N102, N155, and C164 but missing in their corresponding mirror images (Figures 5A and 6A).

To tease out the TPR1-interacting domain, two new constructs were made by dissecting the αE-αF-β2-β3 regions into two halves. C186 contained the N-terminal half of Gα_16 _up to and including the αE-αF regions, while N188 represented its mirror image and contained Gα_16_-specific sequence from the β2-β3 regions onward (Figure 5A). Because these two mirror images contained completely different regions of Gα_16 _and Gα_z_, two distinct anti-Gα_16 _antisera, targeting either the extreme N-terminus (Gα_16_N) or C-terminus (Gα_16_C), were required in the co-immunoprecipitation assay. Although both mutants were expressed to comparable levels (Figure 5B, bottom panels), only N188 (as well as its QL mutant) was co-immunoprecipitated with FLAG-TPR1 (Figure 5B, upper panels). For the reverse co-immunoprecipations, N188 and N188QL, but not C186 and its mutant, were able to interact with TPR1 (Figure 5B, middle panels). Since these findings suggest that only β2-β3 within the αE-αF-β2-β3 region is responsible for TPR1 association, two more chimeras (C174 and N200) were created to split this region into two halves (Figure 5A); C174 possessed Gα_16_-specific β2 region whereas N200 harbored the Gα_16_-specific β3 region. Co-immunoprecipitation experiments with FLAG-TPR1 illustrated that N200 and its constitutively active mutant N200QL interacted with FLAG-TPR1 while C174 and C174QL were ineffective (Figure 5C). Reverse co-immunoprecipitations with anti-Gα_16 _antisera confirmed the ability of TPR1 to associate with N200 and N200QL.

In order to validate the importance of the β3 region of Gα_16 _for TPR1 interaction, N188-C164 and N200-C164 were constructed as shown in Figure 6A. N188-C164 is primarily a Gα_z _backbone with the β2 and β3 regions from Gα_16_, while N200-C164 is essentially Gα_z _with the β3 region made up of the Gα_16 _sequence. Co-immunoprecipitation experiments using anti-FLAG or anti-Gα_z _antisera showed that both chimeras and their respective constitutively active mutants interacted with FLAG-TPR1 (Figure 6B). Association of N200-C164 with FLAG-TPR1 suggests that the β3 region of Gα_16 _alone is sufficient to confer upon Gα_z _the ability to interact with TPR1. On the other hand, replacement of the β3 region of Gα_16 _with the cognate sequence of Gα_z _is expected to disrupt Gα_16_/TPR1 interaction. Two additional chimeras, named zβ3 and zβ2β3, with either the β3 or β2-β3 regions of Gα_z _inserted into a Gα_16 _backbone (Figure 6A) were constructed to test this hypothesis. zβ2β3 and zβ2β3QL failed to co-immunoprecipitate with TPR1 (Figure 6C), thus demonstrating the importance of the β2 and β3 regions of Gα_16 _for interaction with TPR1. However, very weak but detectable associations of zβ3 and zβ3QL with FLAG-TPR1 were observed in co-immunoprecipitation assays (Figure 6C). These results confirm the crucial role of the β3 region for the Gα_16_/TPR1 interaction, and further suggest that the β2 region may facilitate the actions of the β3 strand.

### Activation of Ras via the association of TPR1 with Gα_16 _and its chimeras

**Figure 7:**
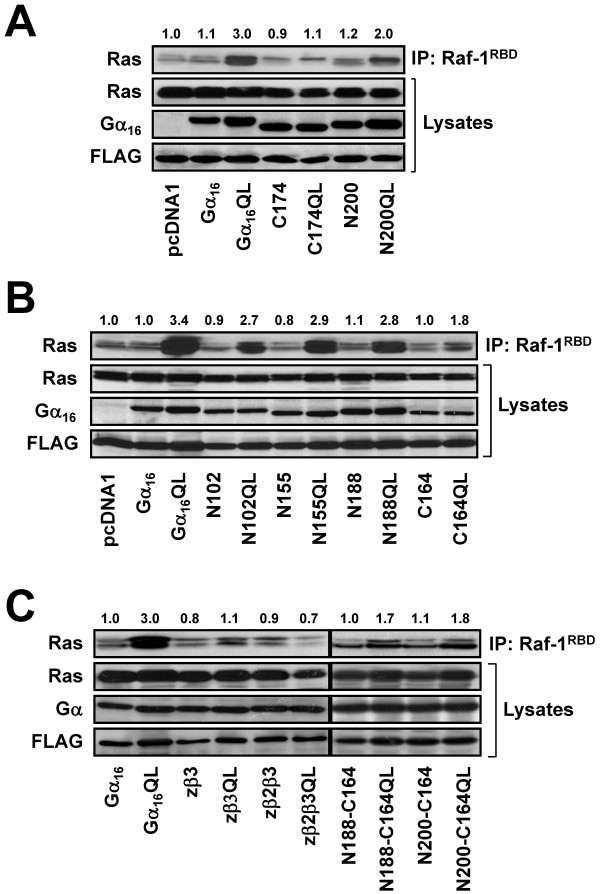
**Activation of Ras by the constitutively active mutants of the TPR1-interacting Gα chimeras**. (A) HEK 293 cells were transiently co-transfected with Ras, FLAG-TPR1 and the wild-type or constitutively active mutants of Gα_16_, C174, N200, pcDNA1 (A), N102, N155, N188, C164 (B), zβ3, zβ2β3, N188-C164, or N200-C164 (C). Cell lysates were immunoprecipitated with GST-bound Ras binding domain of Raf-1 (Raf-1^RBD^) agarose. The cell lysates and the eluted protein samples were subsequently immunoblotted against anti-Ras, anti-FLAG, and anti-Gα_16 _or anti-Gα_z _antibody. The result of the densitometric analysis is shown above the immunoblots and the numerical values represent relative intensities of Ras activity expressed as a ratio of the basal (B), Gα_16_-mediated Ras activity (C) (set as 1.0). Data shown represent one of three sets of immunoblots; two other sets yielded similar results.

Association of TPR1 with Ras may provide a more direct link for Gα_16 _to activate the ERK cascade instead of going through the PLCβ/PKC pathway. If the β3 region of Gα_16 _is essential for functional interaction with TPR1 and Ras, chimeras containing this region should facilitate the activation of Ras while those lacking this region ought to be inactive. As predicted, transfectants expressing N200QL (carrying the β3 region of Gα_16_) exhibited elevated Ras activity whereas C174QL (β3 region from Gα_z_) did not activate Ras (Figure 7A). To extend this study, other TPR1-interacting chimeras were evaluated (Figure 7B). No significant Ras activation was detected when wild-type N102, N155, N188 and C164 were overexpressed. Upon the introduction of the QL mutants of these chimeras, all chimeras induced detectable Ras activation as compared to their corresponding wild-type counterparts (Figure 7B). Likewise, N188-C164 and N200-C164 should be capable of activating Ras because they possess the β3 region of Gα_16_. Indeed, the constitutive active mutants of these chimeras activated Ras (Figure 7C). Conversely, zβ2β3 and zβ3 (the mirror images of N188-C164 and N200-C164, respectively) did not stimulate the Ras activity (Figure 7C). These results indicate that chimeras containing the Gα_16_-specific β3 region possess the ability to activate Ras, and such activity is dependent on the GTP-bound conformation of the chimeras.

### Gα_16_QL-induced Ras activation is independent of PLCβ signaling

The PLCβ-interacting domain of Gα_q _was initially mapped to residues 217-276 [49], corresponding to the α2-β4-α3-β5 regions of Gα_16_. Recent resolution of the crystal structure of Gα_q_-PLCβ has refined the PLCβ-interaction surface on Gα_q _to encompass mainly β2 strand, α2 and α3 helices [50]. Since the putative PLCβ-interacting domain of Gα_16 _is in proximity of the TPR1-interacting β3 region, we asked if activation of Ras and PLCβ can occur independently. The chimeras were transiently expressed in the absence or presence of the G_i_-coupled A_1_R and then assayed for IP formation with or without 10 μM CHA. Predictably, CHA-induced IP formation was observed with transfectants co-expressing Gα_16_, N30, C25, or C44 (Table 2). Chimeras with parts of the PLCβ-interacting regions of Gα_16 _replaced by cognate Gα_z _sequences should exhibit impaired ability to regulate PLCβ. Such chimeras include N246, N266, N295, C272, C219, C186, C174, C164, N188-C164, and N200-C164. Indeed, all ten chimeras failed to stimulate PLCβ in response to CHA (Table 2), although CHA was capable of inhibiting cAMP formation in the transfectants (data not shown). The putative PLCβ-interacting region of Gα_16 _is intact in N102, N155, N188, N200, N210, zβ2β3, and zβ3, and hence these chimeras are expected to support A_1_R-mediated IP formation. However, only transfectants harboring N188, zβ2β3 or zβ3 responded to CHA with a significant increase in IP formation (Table 2). The lack of response to CHA challenge may be attributed to impairment in receptor/G protein recognition. To exclude such a possibility, we tested the ability of the constitutively active mutants of the chimeras to stimulate PLCβ. Those mutant chimeras with the PLCβ-interacting region disrupted or replaced by Gα_z _residues did not exhibit any stimulation of PLCβ, while Gα_16_QL and the previously characterized mutants such as C44 efficiently induced IP formation in the transfectants (Table 2). Among the mutant chimeras with the α2-β4-α3-β5 region intact, only N188QL, N200QL, zβ2β3QL and zβ3QL constitutively stimulated the PLCβ activity (Table 2). The constitutive activity of N200QL suggests that the inability of N200 to mediate A_1_R-induced stimulation of PLCβ may be attributed to defective recognition of receptor. With the exception of N102, N155, and N210, the PLCβ-stimulating abilities of the chimeras were in general agreement with the predicted presence of the putative PLCβ-interacting region.

**Table 2:** Ability of Gα_16/z _chimeras to stimulate IP_3 _production in HEK 293 cells.

*Construct*	*Intact PLCβ Domain*	***A***_***1***_***R-induced IP accumulation***			*QL-induced IP accumulation*
			
		*Basal*	*10 μM CHA*	*Fold Stimulation*	*Fold Stimulation*
pcDNA1	N/A	10 ± 3	11 ± 2	1.1 ± 0.2	N/A
Gα_16_	Yes	23 ± 5	237 ± 13	10.0 ± 0.6*	***7.8 ± 0.4***^***#***^
N30	Yes	23 ± 4	81 ± 10	3.5 ± 0.7*	***6.5 ± 0.4***^***#***^
N102	Yes	17 ± 4	13 ± 4	0.8 ± 0.2	1.1 ± 0.1
N155	Yes	13 ± 3	10 ± 1	0.8 ± 0.1	1.0 ± 0.1
N188	Yes	19 ± 4	155 ± 13	8.2 ± 0.3*	***4.2 ± 0.2***^***#***^
N200	Yes	11 ± 3	15 ± 2	1.3 ± 0.1	***2.0 ± 0.2***^***#***^
N210	Yes	12 ± 3	10 ± 2	0.8 ± 0.2	1.3 ± 0.1
N246	No	10 ± 2	10 ± 1	1.0 ± 0.1	0.8 ± 0.0
N266	No	27 ± 3	17 ± 2	0.6 ± 0.3	1.2 ± 0.2
N295	No	9 ± 1	9 ± 1	1.0 ± 0.1	0.9 ± 0.2
C25	Yes	15 ± 3	92 ± 7	6.1 ± 0.5*	***4.1***^***a***^
C44	Yes	78 ± 11	183 ± 15	2.3 ± 0.2*	***2.6 ± 0.1***^***#***^
C164	No	9 ± 2	8 ± 3	0.9 ± 0.3	1.3 ± 0.2
C174	No	13 ± 3	14 ± 9	1.1 ± 0.1	1.4 ± 0.2
C186	No	4 ± 1	6 ± 2	1.5 ± 0.5	1.4 ± 0.2
C219	No	7 ± 1	9 ± 2	1.3 ± 0.3	1.2 ± 0.1
C272	No	8 ± 1	9 ± 1	1.1 ± 0.1	1.2 ± 0.1
zβ2β3	Yes	21 ± 5	51 ± 2	3.0 ± 0.4*	***4.3 ± 0.4***^***#***^
zβ3	Yes	14 ± 1	79 ± 5	5.6 ± 0.3*	***6.9 ± 0.4***^***#***^
N200-C164	No	15 ± 1	19 ± 1	1.3 ± 0.1	0.9 ± 0.2
N188-C164	No	13 ± 0	14 ± 1	1.1 ± 0.0	1.0 ± 0.2
Gα_z_	No	10 ± 1	9 ± 3	0.9 ± 0.3	1.1 ± 0.1

HEK 293 cells were co-transfected with A_1_R and pcDNA1 or the indicated Gα subunit. Transfectants were labeled with [^3^H]inositol (2.5 μCi/mL) in DMEM containing 5% FBS overnight. IP_3 _formations were examined in the absence (basal) or presence of 10 μM CHA for 60 min. Fold stimulations were calculated as the ratios of CHA-induced to basal IP_3 _accumulations or QL-induced to wild-type IP_3 _accumulations. Data represent the mean ± S.D. of triplicate determinations of a single representative experiment, n = 3; significant responses are shown in bold and italic. Constructs containing an intact putative PLCβ binding domain (α2-β4-α3-β5 regions) of Gα_16 _are marked with a "Yes".

* CHA-stimulated IP_3 _production is significantly greater than basal (DMSO vehicle); paired *t*-test, *p *≤ 0.05.

^***# ***^QL-stimulated IP_3 _production is significantly greater than the corresponding wild-type activity; paired *t*-test, *p *≤ 0.05.

^*a *^QL-induced fold stimulation of 16z25 was extrapolated from [38].

**Figure 8:**
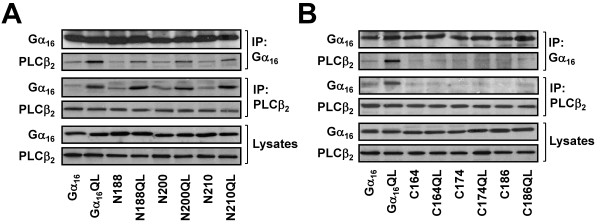
**PLCβ_2 _binds to Gα_16 _via the α2-β4-α3-β5 regions that are distinct from the β3 domain required by TPR1**. (A-B) HEK 293 cells were co-transfected with PLCβ_2 _and the wild-type or constitutively active mutants of Gα_16_, N188, N200, N210 (A), C164, C174, or C186 (B). Cell lysates were immunoprecipitated with anti-Gα_16 _or anti-PLCβ_2 _antisera and analyzed as described in Figure 2. Data shown represent one of three sets of immunoblots; two other sets yielded similar results.

To confirm that the PLCβ-interacting regions of Gα_16_-interaction are distinct from that of TPR1, two series of Gα_16 _chimeras were tested. The first series contained chimeras harboring the putative PLCβ-interacting domains, including N188, N200, and N210. HEK 293 cells were cotransfected with PLCβ_2 _and Gα_16 _or a chimera and then subjected to co-immuniprecipitation using antisera against either Gα_16 _or PLCβ_2_. Interaction between PLCβ_2 _and Gα_16 _was clearly evident, with Gα_16_QL generating a stronger interaction with PLCβ_2 _(Figure 8A). Chimeras N188, N200, and N210 were all capable of being co-immunoprecipitated with PLCβ_2_. Another set of chimeras including C164, C174, and C186 (the mirror images of the first series) was similarly tested (Figure 8B). Due to the replacement of the putative PLCβ-interacting regions with sequences of Gα_z_, these chimeras were expected not to interact with PLCβ_2_. Indeed, even with successful expression of the chimeras and PLCβ_2_, no PLCβ_2 _interaction was detectable for these chimeras (Figure 8B). These results support the notion that the β3 region of Gα_16 _is dispensable for the interaction with PLCβ (e.g. C164).

Since several chimeras can apparently activate Ras through TPR1 despite their inability to stimulate PLCβ, they represent useful tools in delineating complex signaling networks such as those for the regulation of STAT3 and NFκB. If these chimeras can induce the phosphorylation and activation of STAT3 and NFκB, then it would imply that PLCβ activity is not essential. Conversely, a lack of activity on STAT3 and NFκB by these chimeras would indicate that PLCβ action is required. Transfectants co-expressing the chimeras and A_1_R were stimulated with CHA, and the phosphorylation of STAT3 and inhibitor of κB kinase (IKK) monitored by western blot analysis using phosphoprotein-specific antibodies. As shown in Table 3, chimeras with dual TPR1/Ras and PLCβ activating capabilities effectively mediated CHA-induced STAT3 (at Tyr^705 ^and Ser^727^) and IKK phosphorylations, with magnitudes similar to those of Gα_16_. These chimeras included N30, N188, C25, C44, zβ2β3, and zβ3. N200 failed to mediate the CHA-induced STAT3 phosphorylation but supported the IKK phosphorylation (Table 3). TPR1-interacting chimeras which lack PLCβ activity (N102, N155, C164, N188-C164, and N200-C164) were unable to support CHA-induced phosphorylation of STAT3 and IKK (Table 3). Because ERK is required for Gα_16_-mediated STAT3 and NFκB activation [36,37], we further tested the ability of the chimeras to mediate ERK phosphorylation. The profile of ERK phosphorylation mediated by the chimeras closely resembled those for STAT3 and IKK (Table 3). Chimeras lacking both PLCβ and TPR1/Ras activities, including N210, N246, N266, N295, C174, C186, C219, and C272, did not support any of the CHA-induced responses, whereas N30, N188, N200, C25, C44, zβ2β3, and zβ3 mediated CHA-induced ERK phosphorylation (Table 3).

**Table 3:** Gα_16/z _chimera-mediated phosphorylations of STAT3 (both Tyr^705 ^and Ser^727^), IKK, and ERK in HEK 293 cells.

*Construct*	*TPR1/Ras Interaction*	*PLCβ*	*Fold Stimulation of Protein Phosphorylation*			
			
		*Activity*	***P-Tyr***^***705***^***STAT3***	***P-Ser***^***727***^***STAT3***	*P-IKK*	*P-ERK*
pcDNA1	N/A	N/A	1.0 ± 0.0	1.0 ± 0.0	1.2 ± 0.1	1.1 ± 0.1
Gα_16_	Yes	Yes	2.3 ± 0.3*	2.0 ± 0.1*	2.4 ± 0.3*	2.6 ± 0.1*
N30	Yes	Yes	1.7 ± 0.1*	1.7 ± 0.2*	1.7 ± 0.2*	1.9 ± 0.1*
N102	Yes	No	1.2 ± 0.1	1.1 ± 0.1	1.2 ± 0.0	1.3 ± 0.1
N155	Yes	No	1.1 ± 0.0	1.1 ± 0.1	1.2 ± 0.0	1.5 ± 0.0
N188	Yes	Yes	1.7 ± 0.0*	1.4 ± 0.1*	1.9 ± 0.2*	2.2 ± 0.1*
N200	Yes	Yes^*a*^	0.9 ± 0.0	1.1 ± 0.1	1.6 ± 0.2*	1.9 ± 0.1*
N210	No	No	0.9 ± 0.1	0.9 ± 0.0	1.0 ± 0.0	1.1 ± 0.0
N246	No	No	1.1 ± 0.1	1.1 ± 0.1	1.0 ± 0.0	1.1 ± 0.1
N266	No	No	1.4 ± 0.3	1.1 ± 0.0	1.2 ± 0.2	1.3 ± 0.1
N295	No	No	1.0 ± 0.1	0.9 ± 0.0	1.0 ± 0.1	1.1 ± 0.0
C25	Yes	Yes	1.7 ± 0.1*	1.5 ± 0.1*	1.6 ± 0.1*	2.2 ± 0.0*
C44	Yes	Yes	1.6 ± 0.0*	1.4 ± 0.0*	1.5 ± 0.1*	1.8 ± 0.0*
C164	Yes	No	0.9 ± 0.1	1.0 ± 0.1	1.0 ± 0.0	1.3 ± 0.0
C174	No	No	1.1 ± 0.1	0.9 ± 0.0	0.6 ± 0.1	1.2 ± 0.2
C186	No	No	0.9 ± 0.1	1.1 ± 0.2	1.0 ± 0.1	1.3 ± 0.1
C219	No	No	1.1 ± 0.1	1.1 ± 0.2	1.3 ± 0.2	1.2 ± 0.1
C272	No	No	1.0 ± 0.1	0.9 ± 0.1	1.1 ± 0.1	0.8 ± 0.0
zβ2β3	No	Yes	1.6 ± 0.1*	1.5 ± 0.1*	1.5 ± 0.1*	2.2 ± 0.2*
zβ3	No^*b*^	Yes	2.1 ± 0.2*	1.9 ± 0.1*	2.0 ± 0.2*	2.7 ± 0.1*
N200-C164	Yes	No	1.0 ± 0.1	1.1 ± 0.1	0.9 ± 0.1	1.2 ± 0.0
N188-C164	Yes	No	1.0 ± 0.0	0.9 ± 0.1	1.0 ± 0.1	1.2 ± 0.0
Gα_z_	No	No	1.0 ± 0.0	0.9 ± 0.0	1.1 ± 0.0	1.1 ± 0.2

HEK 293 cells were co-transfected with adenosine A_1_R and pcDNA1 or the indicated Gα subunit. Transfectants were serum starved overnight in the presence of 100 ng/mL of PTX and then challenged with 10 μM CHA for 15 min. Cell lysates were then resolved and immunoblotted against various specific antibodies. Results are expressed as fold stimulation over the basal (DMSO vehicle), n = 3; significant responses are shown in bold and italic. Constructs shown to co-immunoprecipitate with FLAG-TPR1 or stimulate PLCβ activity (Table 2) are marked with a "Yes".

* CHA-stimulated phosphorylation of the target protein is significantly greater than the basal; paired *t*-test, *p *≤ 0.05.

^*a *^significant PLCβ activation observed with the QL mutant only (see Table 2).

**Figure 9:**
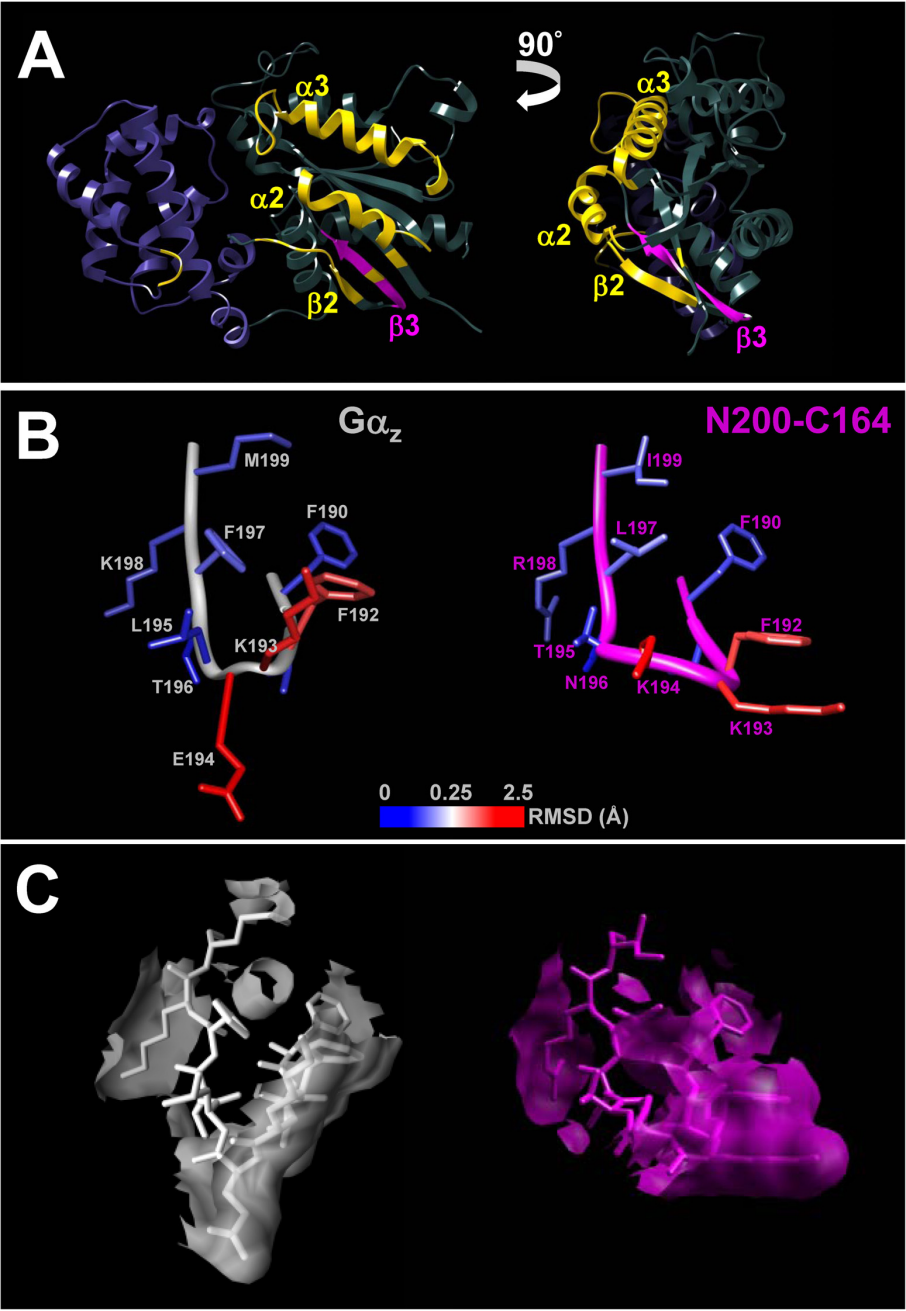
**Molecular modeling of Gα subunits and the predicted conformational changes of the β2/β3 loop**. (A) Left: A molecular model of Gα_16 _is depicted with the helical and GTPase domains highlighted in indigo and dark slate grey, respectively. Regions colored in yellow are the surface-exposing secondary structures that interact with PLCβ, while the TPR1-interacting β3 strand is in magenta. Right: The model is turned 90° clockwise horizontally to show the highlighted residues on α3 helix and the β2-β3 loop in a different orientation. (B) Models of Gα_z _(white) and N200-C164 (magenta) for the predicted conformational changes by mutating the β2-β3 loop of Gα_z _are depicted in tube-like carbon backbone. Side chains of the residues are colored according to the root mean square deviations (RMSD) between these residues on the two models. Blue and red colors represent the least and greatest deviations, respectively. The scale bar shows the corresponding RMSD range. (C) The β2-β3 loop of Gα_z _and N200-C164 are shown in the same orientations as in (B) but with the carbon backbone, side chains and the accessible molecular surfaces in the same colors to visualize the overall deviations in molecular surfaces and volumes caused by the mutations.

^*b *^Very weak association with TPR1 was detected in co-immunoprecipitation assays (Figure 9).

Finally, we employed luciferase reporter gene assays to demonstrate transcriptional regulation of STAT3 and NFκB by those chimeras that possess dual TPR1/Ras and PLCβ activating capabilities. In agreement with our previous studies [36,37], CHA induced STAT3- and NFκB-driven luciferase activities in transfectants co-expressing Gα_16 _but not Gα_z _(Table 4). N30, N188, N200, C25, C44, and zβ3 chimeras all supported CHA-induced STAT3- and NFκB-driven luciferase activities, whereas no transcriptional activation was observed in transfectants co-expressing chimeras that failed to mediate STAT3, IKK, and ERK phosphorylations (Table 4). Collectively, these studies demonstrate that dual TPR1/Ras and PLCβ activating capabilities of Gα_16 _may be essential for its regulation of complex signaling networks such as those for the activation of STAT3 and NFκB.

**Table 4:** Gα_16/z _chimera mediated STAT3-driven and NFκB-driven luciferase activities.

*Construct*	*STAT3-driven Luciferase Fold Stimulation*	*NFκB-driven Luciferase Fold Stimulation*
pcDNA1	1.0 ± 0.0	1.1 ± 0.1
Gα_16_	2.4 ± 0.1*	3.0 ± 0.2*
N30	1.7 ± 0.2*	2.1 ± 0.2*
N102	0.9 ± 0.1	1.3 ± 0.1
N155	1.0 ± 0.0	1.3 ± 0.0
N188	1.9 ± 0.2*	1.9 ± 0.2*
N200	1.5 ± 0.0*	1.7 ± 0.1*
N210	1.2 ± 0.1	1.2 ± 0.1
N246	0.9 ± 0.1	1.2 ± 0.1
N266	1.1 ± 0.1	1.2 ± 0.1
N295	1.2 ± 0.2	1.1 ± 0.1
C25	1.8 ± 0.1*	2.0 ± 0.1*
C44	1.6 ± 0.0*	1.9 ± 0.2*
C164	1.2 ± 0.0	1.3 ± 0.2
C174	1.2 ± 0.1	1.2 ± 0.1
C186	1.2 ± 0.0	1.3 ± 0.1
C219	1.3 ± 0.2	1.2 ± 0.1
C272	1.1 ± 0.1	1.2 ± 0.1
zβ3	1.7 ± 0.2*	2.4 ± 0.0*
N200-C164	1.2 ± 0.1	1.0 ± 0.1
N188-C164	1.2 ± 0.1	1.3 ± 0.0
Gα_z_	1.1 ± 0.0	1.2 ± 0.0

HEK 293 cells were co-transfected with adenosine A_1_R, pSTAT3-luc/pNFκB-luc, and pcDNA1 or the indicated Gα subunit. Transfectants were serum starved for 4 h in the presence of 100 ng/mL of PTX and then challenged with 10 μM CHA overnight. Cell lysates were analyzed for luciferase activity. Data represent the mean ± S.D. of triplicate determinations, n = 3; significant stimulations are shown in bold and italic. * CHA-stimulated protein phosphorylation is significantly greater than the basal; paired *t*-test, *p *≤ 0.05.

## Discussion

Protein-protein interactions are central to the functions of Gα subunits and each Gα subunit has to coordinate such interactions in a timely manner according to its guanine nucleotide binding state. Once activated, the GTP-bound Gα subunit has a limited time-span to regulate downstream effectors before its intrinsic GTPase activity turns it back to the inactive GDP-bound state, a process which can occur rapidly in the presence of RGS proteins. Hence, simultaneous regulation of multiple signaling pathways by activated Gα_16 _is desirable but this entails the deployment of different binding surfaces. Although Ras can be activated indirectly by PLCβ signaling, our results provide a structural basis for Gα_16 _to stimulate Ras through interaction with TPR1 instead of PLCβ. Moreover, functional analyses of Gα_16/z _chimeras reveal that dual stimulation of TPR1/Ras and PLCβ may be essential for Gα_16 _to activate downstream transcription factors such as STAT3 and NFκB.

The extensive array of Gα_16/z _chimeras has enabled us to pinpoint the TPR1-interacting domain to the β3 region (and also possibly β2 as well) of Gα_16_. The β2-β3 region lies between the Switch I (mainly β2 strand) and II (mainly α2 helix) regions [56] and is accessible for protein-protein interaction (Figure 9A). Since activation of the Gα subunit alters the conformation of several switch regions drastically, proteins like PLCβ that bind to the Switch regions will interact with the GTP-bound active state much effectively than the GDP-bound basal state of the Gα subunit (Figure 8). However, the differences of the interactions between TPR1 and Gα_16_WT or Gα_16_QL are comparably smaller, and TPR1 can obviously bind to Gα_16_WT. Such observation implies that TPR1 may bind to Gα_16 _at regions with relatively less drastic conformational changes. Our results suggested that the β3 strand of Gα_16 _alone appears to be sufficient for the interaction with TPR1. The mere incorporation of the β3 region of Gα_16 _confers upon Gα_z _the ability to bind TPR1 (N200-C164 chimera). N200-C164 has the amino acid sequence ^201^KTNLRIVDVG from Gα_16 _inserted into a Gα_z _backbone, but only the first six residues are different from the corresponding Gα_z _sequence (^194^ELTFKMVDVG). Based on the crystal structure of Gα_i1 _[43], molecular modeling of Gα_z _reveals that Glu^194 ^is located at the "hook-shaped" β2-β3 turn (Figure 9B), which is often composed of two oppositely charged residues flanked by two interacting hydrophobic residues except Gα_16_, with a threonine following the Lys^201^. The predicted β2-β3 turn of N200-C164 showed a more widened conformation (Figure 9B-C), which presumably forms a characteristic microdomain on Gα_16 _for TPR1 interaction. The fact that the chimera zβ3 exhibited residual binding to TPR1 (Figure 6C) suggested that other regions like the β2 strand may also participate in the binding of TPR1 because its removal in the zβ2β3 chimera can further suppress TPR1 binding as compared to zβ3 (Figure 6C). Since several chimeras failed to generate a response in all of the functional assays, these chimeras might not be able to adopt the active conformation properly and it remains possible that additional residues other than the β3 region may bind to TPR1.

Although the chimeras used in the present study were not tailored for the mapping of the PLCβ-interacting domain of Gα_16_, they nonetheless proved useful in locating the overall site for the binding of PLCβ. As in the case of Gα_q _[49,50], the regions comprising of β2, β3, α2, and α3 are likely to form the PLCβ-interacting surface of Gα_16 _(Figure 1) because chimeras with one or more of these regions disrupted all failed to stimulate IP formation (Table 2). A more interesting observation is that chimeras including N102, N155, N200, and N210 could not activate PLCβ at all, and only the constitutively active form of N200 could stimulate PLCβ weakly, whereas the chimera N188 recapitulates the PLCβ-activating capability. N102 and N155 contained all the putative PLCβ-interacting domains and yet could not activate PLCβ, albeit both interacted with TPR1 and activated Ras. One plausible explanation is that both chimeras have a hybrid helical domain (αA-αG; Figure 3A) which may affect its structural and functional integrity. Indeed, all Gα_16/z _chimeras that can stimulate PLCβ activity (e.g., C44 and N188) contain a contiguous helical core (αA-αF helices) in addition to the putative PLCβ-interacting domains. An early study of Gα_s _suggested that its helical domain could function as an internal GAP for the GTPase domain [57], and the intra-molecular interaction between the GTPase and helical domains was proven to be essential for guanine nucleotide binding and receptor-mediated activation [58,59]. Furthermore, the αC-αD loop of Gα subunits has been recognized as Switch IV which shows significant conformational changes in different guanine nucleotide-binding states and could reduce the nucleotide exchange rate when mutated [60]. It is therefore possible that a hybrid helical domain may impede the activation of PLCβ by the Gα_16/z _chimeras. The sudden regain of PLCβ-activating property of N188 which contains a helical domain completely derived from Gα_z _also implies that structural integrity of the helical domain is critical for PLCβ activation. As revealed in the very recent crystal structure of Gα_q_-PLCβ3 [50], the β2 and β3 strands of Gα_q _interact extensively with the C2 domain of PLCβ3, and these two strands also heavily contribute to the overall scaffold of the GTPase domain. Replacement of one or both of them, as in the N200 and N210 chimeras, might disrupt PLCβ interaction severely. Nonetheless, N200 can successfully form a heterotrimer with FLAG-tagged Gβγ dimer in co-immunoprecipitation experiment (unpublished data), excluding the possibility of improper folding of the chimera.

A molecular model of Gα_16 _is constructed by homologous modeling based on the crystal structure of Gα_q _to visualize the potential interacting surfaces for PLCβ and TPR1. Except for the unusually long α4-β6 loop and differences in the N-terminal helix (not shown in the model), Gα_16 _basically fit very well to the structure of Gα_q_. Assuming that Gα_16 _utilizes domains similar to those of Gα_q _for the binding of PLCβ [49], it is entirely feasible for Gα_16 _to simultaneously regulate PLCβ and TPR1. As shown in the right panel of Figure 9A, the critical PLCβ-interacting residues are clustered mostly on the left hand side of the GTPase domain of Gα_16 _(yellow-colored), while the putative TPR1-interacting β3 region is located in the lower quadrant of the right hand side without severe overlap with the PLCβ-interacting region. Preliminary co-immunoprecipitation studies indeed suggest the existence of Gα_16_/PLCβ and PLCβ/TPR1 complexes (unpublished data). Further studies will be required to confirm if a Gα_16_/PLCβ/TPR1 complex truly exists. TPR motif-containing adaptors such as Rapsyn are known to cluster signaling molecules for the efficient propagation of signals [61]. It is conceivable that TPR1 may serve a similar function in G protein pathways. Its lack of association with Gα_13_, Gα_t1 _[6] and Gα_z _(Figure 2B) suggests that TPR1 can selectively link G protein signals to Ras-dependent pathways. For those that can interact with TPR1, it remains to be determined if binding to TPR1 confers upon them the same repertoire of signaling capabilities.

The distinct locations for PLCβ- and TPR1-interacting regions infer that Gα_16 _can regulate them independently. Indeed, we have demonstrated that some chimeras (e.g., N102, N155, C164, N188-C164, and N200-C164) can bind TPR1 (Figures 3, 4, and 6) and induce Ras activation (Figure 7) even though they lack the ability to stimulate PLCβ (Table 2). This raises an interesting possibility that, depending on the composition of the signaling modules within a cell, activation of Gα_16 _may differentially regulate TPR1/Ras and PLCβ signaling pathways. Both Ras and PLCβ activities are apparently required for the regulation of STAT3 and NFκB [36,37] , but it is not clear if the two components are arranged in parallel or in series. Given that the activation of STAT3 and NFκB could only be detected with chimeras possessing the ability to activate both Ras and PLCβ, it would seem that the two pathways are independently required for the regulation of STAT3 and NFκB. The need for multiple input signals increases signaling fidelity and specificity as well as ensuring a stringent control of transcription.

The possibility of Gα_16 _to bind PLCβ and TPR1 simultaneously raises some interesting questions regarding the fidelity of G protein signals. One concern is whether the two signaling pathways can be regulated independently. Stimulation of PLCβ by GPCR can be mediated via members of the Gα_q _subfamily [62,63] or through the Gβγ dimer [64,65], with the latter restricted primarily to some isoforms of PLCβ. Like Gα_16_, both Gα_q _[6] and Gα_14 _(unpublished data) can interact with TPR1. Since many ligands that act on G_q_-coupled receptors are mitogenic [10,66], linkage through Gα_q_/TPR1 provides a means for the efficient stimulation of the Ras/Raf/MEK/ERK cascade for cell proliferation. Given that both PLCβ and TPR1 are ubiquitously expressed, the ability of Gα_16 _as well as other Gα_q _subfamily members to selectively activate one of the two pathways may have to rely on alternative means of signal segregation, such as spatial orientation [67], formation of macromolecular signaling complexes [68], and compartmentalization of signaling components [69]. Attachment of the Gα_q _subunit to the lipid bilayer [70], as well as its targeting to plasma membrane microdomains and to intracellular organelles [71], have been shown to affect Gα_q _signaling. It should also be noted that the presence of p63RhoGEF can affect the binding of PLCβ and TPR1 to Gα_16 _and differentially inhibit their signaling [25]. The p63RhoGEF-interacting domain on Gα_16 _has yet to be elucidated, but is expected to encompass the α2-β4-α3-β5 regions and the C-terminal α5 helix based on the crystal structure of Gα_q_-p63RhoGEF-RhoA complex complex (Figure 1) [13]. In this regard, the Gα_16/z _chimeras represent useful tools to confirm such a prediction.

## Conclusions

This study provided evidence for the importance of the β3 strand of Gα_16 _for the interaction with TPR1 and subsequent activation of Ras, but the β3 strand appears to be dispensable for PLCβ interaction. The integrities of both helical and GTPase domains are essential for PLCβ activation. Gα_16 _can signal through TPR1/Ras and PLCβ simultaneously and independently to regulate transcriptional events involving STAT3 and NFκB by utilizing different structural domains to bind TPR1 and PLCβ. Overall, Gα_16 _is able to interact with multiple molecular partners to convey different streams of signal transduction.

## List of abbreviations used

A_1_R: type 1 adenosine receptor; AGS: activator of G protein signaling; CHA: *N*^6^-cyclohexyladenosine; FBS: fetal bovine serum; G protein: heterotrimeric guanine nucleotide-binding proteins; GPCR: G protein-coupled receptor; GTPase: GTP hydrolase; IKK: inhibitor of κB kinase; IP_3_: inositol trisphosphates; NFκB: nuclear factor κB; PLCβ: phospholipase Cβ; PTX: pertussis toxin; RGS: regulator of G protein signaling; STAT3: signal transducer and activator of transcription 3; TPR1: tetratricopeptide repeat 1

## Authors' contributions

AMFL and RKHL performed most biological experiments. EXG assisted in experiments. MKCH performed homology modeling. AMFL, MKCH and YHW conceived and designed the experiments and wrote the manuscript. RDY participated in the design of the study. All authors read and approved the final manuscript.
